# Contributing factors for a satisfying orofacial pain patient journey: a mixed-methods study

**DOI:** 10.22514/jofph.2025.050

**Published:** 2025-09-12

**Authors:** Hedwig A. van der Meer, Annemarie van der Wal, Annemiek Rollman, Frank Lobbezoo, Corine M. Visscher

**Affiliations:** ^1^Department of Orofacial Pain and Dysfunction, Academic Centre for Dentistry Amsterdam (ACTA), University of Amsterdam and Vrije Universiteit Amsterdam (VU Amsterdam), 1081LA Amsterdam, The Netherlands; ^2^SOMT University of Physiotherapy, 3821BN Amersfoort, The Netherlands; ^3^Department of Rehabilitation Sciences and Physiotherapy, Faculty of Medicine and Health Sciences, University of Antwerp (UA), 2610 Antwerp, Belgium; ^4^Educational Directorate, Department of Public Health, Academic Centre for Dentistry Amsterdam (ACTA), University of Amsterdam and Vrije Universiteit Amsterdam (VU Amsterdam), 1081LA Amsterdam, The Netherlands; ^5^Department of Orofacial Pain and Jaw Function, Faculty of Odontology, Malmö University, 214 21 Malmö, Sweden

**Keywords:** Orofacial pain, Patient journey, Healthcare, Dentistry, Temporomandibular disorders, Multidisciplinary treatment

## Abstract

**Background**: Due to the many different underlying disorders causing orofacial pain, management of orofacial pain is a challenge. Many patients seek help from different healthcare providers during their patient journey. The primary aim of this study was to describe the physical and emotional patient journey of patients with orofacial pain. The secondary aim was to determine which personal factors may have contributed to number of healthcare providers visited and patient journey satisfaction. **Methods**: For this, a concurrent triangulation mixed methods design was used. Patients 18 years or older with orofacial pain who visited the Orofacial Pain and Dysfunction (OPD) clinic of the Academic Centre of Dentistry Amsterdam were recruited. Participants filled out questionnaires regarding their patient journey, locus of control, readiness to change, catastrophizing, illness perception, and kinesiophobia. A linear regression analysis was performed to determine which factors were associated with the number of healthcare providers visited and the patient journey satisfaction. A subsample of participants was invited to an additional in-depth interview about their patient journey experiences. **Results**: A total of 102 participants visited an average of 2.7 healthcare providers before their visit to the OPD clinic. There was an association between patient satisfaction and readiness to change as well as internal locus of control, and between the number of healthcare providers visited and readiness to change, as well as illness perception and chance-related locus of control. Themes related to a satisfactory patient journey included clear communication, feeling taken seriously, and thinking along with the patient. Non-helpful factors were long wait times, financial factors, perceived lack of knowledge of the healthcare providers, and feeling helpless. **Conclusions**: The journey of patients with orofacial pain is complex, and the patient satisfaction could be improved by improving the efficiency and clear communication to patients.

## 1. Introduction

Approximately one in five patients worldwide are affected by chronic pain [1]. One of the most common chronic pain complaints described in the International Classification of Diseases (ICD-11), are “chronic primary or secondary headache, or orofacial pain” [2]. The diagnostic criteria for these pain complaints are described in the International Classification for Orofacial Pain (ICOP) [3]. Orofacial pain can be attributed to disorders of dentoalveolar and anatomically related structures (*e.g.*, teeth and gums), masticatory muscles or joint disorders, nerve lesions, or are idiopathic in nature [3]. Dental pain is the most common cause for orofacial pain and is managed by the dentist. However, when orofacial pain is not caused by dental issues, it can be more difficult to identify the underlying problem and to then refer the patient accordingly.

Orofacial pain is often managed by a specialized dentist. After dental pain, temporomandibular disorders (TMDs) are the most common cause for orofacial pain not counting oncology-related pain [4]. Furthermore, orofacial pain can originate from other sources, like problems originating in the ear, nose or throat, a lesion or disease of the cranial nerves, or it could be idiopathic in nature [3]. Differential diagnostics can be challenging when orofacial pain resembles other disorders such as primary headaches, and adequate knowledge of different causes of orofacial pain and their clinical representation is needed [3, 5]. Besides the challenge in the diagnostic process, there lies a challenge in identifying important factors that may contribute to the development of chronic complaints, and addressing these accordingly [2, 6]. When these complex orofacial pain complaints are present, interprofessional collaboration is often advised [7].

Current literature shows that an important motivation for patients with orofacial pain to seek help is a need for information about their complaints [8]. They need to receive this information from a professional to be reassured that what they are doing is correct and will not make their pain worse [8]. They also feel the need to be validated of their pain by receiving a proper diagnosis [8]. In addition, higher pain intensity and fear of jaw movement are reported to be related to the patients’ decision to seek care [9]. Due to the complexity of orofacial pain, patients have often seen many other health care providers before receiving a multidisciplinary orofacial treatment. In a previous retrospective study, patients with orofacial pain complaints had seen an average of three different disciplines (including general medical practitioners, general dentists, maxillofacial surgeons, or ear- nose- and throat (ENT) doctors) before coming to a specialized clinic [10]. As there are many possible causes for orofacial pain, there may be a need to visit different disciplines to reach the correct diagnosis and treatment plan. However, patients may be unaware where they can go to for help, and individual health care providers may not have enough experience with these issues to refer the patient to the most appropriate specialized health care provider [11, 12].

Clearly, various factors may play a role in the individual patient’s journey, such as personal (psychosocial) factors and knowledge of the specialties involved in orofacial pain. A promising tool to plot the various factors involved in the patient journey of patients with orofacial pain is the so called “patient journey mapping” [13]. This method does not only look at quantitative data, such as the number of healthcare providers visited (physical journey), but also at the patients’ experiences (emotional journey). Therefore, the primary aim of this study was to describe the physical and emotional “patient journey” of patients with orofacial pain. The secondary aim of this study was to determine which personal factors may have contributed to the number of healthcare providers visited and patient journey satisfaction. For both aims, a mixed method will be used.

## 2. Materials and methods

This manuscript is structured according to the “Good Reporting of a Mixed Methods Study” (GRAMMS) [14]. For the qualitative part, the Standards for Reporting Qualitative Research (SRQR) are followed [15]. The funders of this study played no role in the design, conduct or reporting of the study. The study was approved by the Ethics Committee of the Academic Centre of Dentistry Amsterdam (ACTA) (2021-77518).

### 2.1 Study design

A concurrent triangulation mixed methods design was used [16]. For the primary research aim, quantitative (physical journey) and qualitative (emotional journey) data were collected. Because a qualitative design allows for a deeper understanding of emotions and experiences, a mixed-methods study is most effective for a complete patient journey mapping. For the secondary research question, only quantitative data were used.

### 2.2 Study population

Patients were recruited from the Orofacial Pain and Dysfunction (OPD) clinic of ACTA. In this clinic, patients with orofacial pain or dysfunction complaints, bruxism, or sleep apnea are managed by a multidisciplinary team consisting of dentists specialized, or in training to be specialized, in orofacial pain, orofacial physiotherapists, and a psychologist. The dentists specialized in orofacial pain have completed a three-year fulltime postgraduate program, which is a combination of a theoretical and clinical program to deepen their knowledge and experience with the various types of patients with orofacial pain. Before the intake visit, all patients fill out an online questionnaire which includes the Symptom Questionnaire of the Diagnostic Criteria for TMD (DC/TMD) [17], as well additional questionnaires regarding their complaints, co-morbid conditions such as headache [18], sleep, and psychosocial factors. During the intake, a standardized examination is conducted by a trained dentist according to the DC/TMD protocol [17].

Patients were included in the study if they (1) reported orofacial pain complaints; (2) were minimally 18 years old; (3) were able to communicate in Dutch; and (4) signed an informed consent form. There were no exclusion criteria based on specific types of orofacial pain. Patients were invited by one of the healthcare providers of the OPD clinic between March 2022 and June 2023. A random subgroup of participants was invited to participate in an in-depth interview. From the list of included participants, every fifth person was invited to participate in the interviews. If one person declined, the next person was invited. Recruitment for the interviews continued until saturation was achieved, which was considered to be the case when no new information was identified from the last two interviews. A priori, this was expected to occur after six to twelve interviews [19].

### 2.3 Quantitative data collection: characteristics and outcome measures

The following participant characteristics were extracted from the participants’ medical files: age, gender, diagnosis, orofacial pain intensity, interference, and duration. The orofacial pain intensity and interference were extracted from the Graded Chronic Pain Scale, which resulted in a Characteristic Pain Intensity Score (0–100) and an interference score (0–100) [20]. Additionally, information regarding headache, oral behaviors, somatic symptoms, depression, anxiety, stress, and optimism were also extracted from the medical files.

All participants received an additional survey specifically designed for this study, which included: (1) questions regarding their orofacial pain complaints (what are the complaints, duration, where did they seek information about the complaints); (2) questions regarding the number of, and experience with, healthcare practitioners visited prior to their visit at the OPD clinic for their orofacial pain complaints; (3) the Multidimensional Health Locus of Control scale (MHLCS) [21]; (4) the Multidimensional Pain Readiness to Change Questionnaire (MPRCQ) [22]; (5) the Pain Catastrophizing Scale (PCS) [23]; (6) the brief Illness Perception Questionnaire (IPQ) [24]; and (7) the Tampa Scale for Kinesiophobia for TMD (TSK-TMD) [25]. The survey was self-administered by the participants in their home environment. The Dutch versions of the MHLCS, MPRCQ, PCS and IPQ are downloadable through the website www.meetinstrumentenzorg.nl.

#### 2.3.1 The multidimensional health locus of control scale (MHLCS)

Locus of control was measured using the MHLCS [21], which was available and validated in Dutch [26]. The MHLCS consists of 18 questions and results in three outcomes related to the type of locus of control: internal, external (doctor), or chance. The scores range from 6 to 36 for each type of locus of control. A higher score indicates a higher degree of that type of locus of control being present. Even though the MHLCS has not been validated specifically for patients with orofacial pain, it is found to be valid and reliable among people with various pain conditions [27].

#### 2.3.2 The multidimensional pain readiness to change questionnaire (MPRCQ)

Motivation or readiness to change was measured using the Dutch version of the MPRCQ2-26 [22, 28], which consists of 26 items and nine subscales. The total score of the questionnaire was used for this study, which is the sum of the nine subscales and can range from 9 to 63. A higher score indicates more motivation to change. Currently, there are no clinimetric measures available for patients with orofacial pain, but overall the MPRCQ2-26 shows good validity in patients with rheumatic diseases [29].

#### 2.3.3 The pain catastrophizing scale (PCS)

Pain catastrophizing was measured using the validated Dutch version of the PCS [23, 30]. This questionnaire consists of 13 items regarding rumination, magnification, and helplessness. For the current study, the total score of all items (range 0 to 52) was used for the analysis, where a higher score implies higher levels of catastrophizing. The reliability of the PCS is moderately acceptable and the concurrent validity adequate [31].

#### 2.3.4 The brief illness perception questionnaire (IPQ)

In this 9-item questionnaire, illness perception is measured [32]. This questionnaire is commonly used in pain clinics for evaluation purposes and there is no official calculation for the total score. For the current study, the total score of the first 8 items was calculated, where each item could receive a score from 0 to10, resulting in a total score range of 0 to 80. A higher score reflects a more negative illness perception. The last item is an open-ended question and was therefore not part of the scoring. The brief IPQ shows moderate overall test-retest reliability in patients with musculoskeletal disorders [33], and overall good clinimetric properties for patients with a wide range of illnesses [34] and is validated in Dutch [32].

#### 2.3.5 The Tampa scale for kinesiophobia for TMD (TSK-TMD)

Fear of movement as related to jaw complaints was assessed using the TSK-TMD [25], which is validated in Dutch for patients with TMD. This questionnaire consists of 18 items and generally has good reliability and convergent validity [25]. The total score (range 17 to 68) is the sum of all items, and a higher score indicates higher levels of kinesiophobia.

### 2.4 Qualitative data collection: interviews

Semi-structured interviews were performed with participants using a pre-defined topic guide (**Supplementary Table 1**), asking in-depth questions about the answers to the survey they filled out regarding their experience with health care providers. The questions aimed to identify the “why” and the “why continue” behind seeking help from different specialists, as well as their personal experience of their emotional journey.

The participants were interviewed by one researcher (HvdM) and the interviews were analyzed by two researchers independently (HvdM and AvdW). Two other researchers (AR and CV) had an advisory role regarding the topic guides and analysis. HvdM is an orofacial physiotherapist at the OPD clinic and knew some of the participants personally. The interviews were held either at the OPD clinic or online in an environment where the patient was comfortable and not disturbed. The interviews were audio- and videorecorded, transcribed verbatim, and imported in Atlas.ti version 23.0 (ATLAS.ti Scientific Software Development GmbH, Berlin, BE, Germany) for further analysis.

### 2.5 Data analysis

Descriptive statistics were used to describe the participants’ characteristics and outcome measures. The summary figures were created with the online program Canva (www.canva.com).

#### 2.5.1 Primary research question: physical and emotional patient journey

The physical patient journey was described using numerical or descriptive data: number of healthcare professionals visited before ACTA, type of healthcare professionals visited before ACTA, reasons to continue seeking care (*e.g.*, no personal connection, healthcare provider could not help any further, healthcare provider referred to someone else), and overall satisfaction on a Likert scale from 0 (not satisfied) to 5 (extremely satisfied).

To describe the emotional patient journey, qualitative data analysis was used. Open coding was applied to identify important aspects of the interviews using Atlas.ti. These codes were compared after the third and last interview, checking if consensus between researchers (HvdM and AvdW) was present. If there was no consensus, the researchers discussed the codes until consensus was reached. Then, axial coding was applied to identify themes. The themes were presented to participants via e-mail, and they were asked if they identified themselves with these themes, to increase credibility [35]. The emotional journey was then described based on results from the interviews, using the identified themes.

Finally, the results of the physical journey were combined with those of the emotional journey, to depict the full patient journey (triangulation of results).

#### 2.5.2 Secondary research question: factors contributing to patient journey

There were two dependent variables for the secondary research question related to the patient journey: (1) the number of healthcare providers visited before ACTA, and (2) the overall patient satisfaction; both scored on a 5-point Likert scale (1 not satisfied to 5 extremely satisfied).

For each of these two outcome measures, a linear regression analysis, with a forward approach, was conducted to determine which factors contributed to the patient journey. The following personal factors were included as possible predictors in the models: internal-, external- or chance-related locus of control, readiness to change, catastrophizing, illness perception and kinesiophobia. Before the predictors were added to the model, the assumptions for linear regression (linear relationship, independence, homoscedasticity, and normality) were checked [36]. Analysis of the data was performed using the Statistical Package for the Social Sciences (IBM SPSS) version 26.0 (SPSS Corp, Chicago, IL, USA). Findings were considered significant when the *p*-value was < 0.05.

## 3. Results

### 3.1 Participants’ characteristics

During the period of March 2022 to June 2023, a total of 595 new patients were seen at the OPD clinic. Of these, 216 patients did not meet the inclusion criteria, 140 patients were not informed about the study and 35 did not consent to participation. Thus, 204 patients agreed to participate and signed the informed consent form. Of these, 102 completed the additional questionnaire and were therefore included in the data analysis. Descriptives for the included participants are depicted in Table 1. For the interviews, 25 participants were invited, of which 10 were interviewed after additional consent was signed.

**Table 1.** Patient characteristics (age, gender, orofacial pain diagnoses and personal factors) for the total population.

	Total Population (N = 102)
Age; mean ± SD (range; min–max)	46.0 ± 15.9 (18–77)
Gender, N (%)	
● Female	86 (84.3)
● Male	16 (15.7)
DC/TMD diagnosis, N (%)	
● Myalgia	68 (66.7)
● Arthralgia	32 (31.4)
● ADD with reduction	13 (12.7)
● ADD without reduction	2 (2.0)
● Hypermobility	2 (2.0)
● Internal joint disorder	4 (3.9)
Self-reported headache	
● Yes	67 (65.7)
● No	35 (34.3)
Headache diagnosis	
● Headache attributed to TMD	20 (19.6)
● Migraine	21 (20.6)
● TTH	54 (52.9)
Time with orofacial pain	
● <3 mon	13 (12.7)
● 3–6 mon	8 (7.8)
● 6–12 mon	9 (8.8)
● 1–5 yr	37 (36.3)
● 5–10 yr	12 (11.8)
● 10 yr	15 (14.7)
● Missing	8 (7.8)
Personal factors	
● Health Locus of Control	
○ Internal	20.1 ± 4.5
○ External	24.7 ± 4.8
○ Chance	24.2 ± 4.3
● Illness Perception, IPQ; mean ± SD	45.8 ± 9.7
● Kinesiophobia TSK; mean ± SD	34.4 ± 7.8
● Readiness to change; mean ± SD	42.4 ± 7.9
● Catastrophizing; mean ± SD	
○ Rumination	7.9 ± 4.3
○ Magnification	3.6 ± 2.8
○ Helplessness	9.0 ± 5.4
○ Total score	20.5 ± 10.9

*N: number of participants; SD: standard deviation; TMD: temporomandibular disorder; TTH: Tension-type headache; IPQ: illness perception questionnaire; TSK: Tampa scale for kinesiophobia; DC: Diagnostic Criteria; ADD: Anterior Disc Displacement.*

The age of the included participants (N = 102) ranged from 18 to 77, with a mean of 46 years and standard deviation (SD) of 15.9. The majority (84.3%) was female, and myalgia was the most common OFP diagnosis (66.7%), followed by arthralgia (31.4%). Almost two-thirds of the participants also experienced headaches (65.7%). Tension-type headache (TTH) was the most common type of headache (52.9%), followed by migraine (20.6%), as based on the Headache Screening Questionnaire [18]. Lastly, headache attributed to TMD, as diagnosed with the DC/TMD, was present in 19.6% of the people with headaches [17]. The subgroup of participants who was interviewed (N = 10) was mostly representative of the total population, as their mean age was 49 years and 60% were female.

### 3.2 Primary aim: description of physical patient journey: quantitative results

For most patients, the complaints started with pain in the facial region (64.7%) and/or head (49.0%). Other complaints at the start of the patient journey were joint sounds (37.3%), limited mouth opening (33.3%), teeth wear (18.6%), and/or sleep problems (2.9%). The patients reported that they had gathered information about their complaints online (27.5%), from family or friends (6.9%), or directly from a healthcare provider (60.8%). The healthcare provider that was most frequently visited for information was the general dentist (19.6%), followed by the general medical physician (15.7%) and (medical) specialists (15.7%).

Participants visited between 1 and 9 healthcare providers for their complaints, with an average of 2.7 healthcare providers before their visit to the OPD clinic. Overall, a total of 39 participants (38.2%) indicated that they were satisfied with their patient journey (score 4 satisfied or 5 extremely satisfied). *Post-hoc* analysis using a Pearson correlation test showed a small but significant negative association between the number of healthcare providers visited and patient satisfaction (*r* = −0.264, *p *= 0.007). A *post-hoc* Spearman’s test also showed a small but significant association between the number of healthcare providers visited and the duration of the complaints (*r* = 0.230, *p *= 0.040).

The healthcare provider visited first was most frequently a general dentist (49.0%), followed by an orofacial physiotherapist (16.7%). Of the 58 participants who visited a second healthcare provider and the 42 participants who visited a third, the general dentist, dentist specialized in orofacial pain, and orofacial physiotherapist were most frequently visited. Fig. 1 depicts the beginning of the patient journey as a graphical summary. For details of the healthcare providers visited, see **Supplementary Table 2**.

**Fig. 1.** Graphical summary of the beginning of the physical and emotional patient journey of patients with orofacial pain. The five steps of the beginning of the patient journey are shown: complaints, seeking help, top 5 first healthcare providers visited, experiences with the first healthcare provider and reasons to continue seeking care. TMJ: temporomandibular joint; OFP: orofacial pain.

### 3.3 Primary aim: description of physical patient journey: qualitative results

The analysis of the first nine interviews resulted in 13 themes, which are all connected to the patient journey experience. Fig. 2 depicts the schematic overview of the patient journey, where the identified themes can be divided into factors contributing to a positive patient journey, factors contributing to a negative patient journey, and perspective on multidisciplinary approach.

**Fig. 2.** Overview of themes identified related to the patient journey experience based on the qualitative analysis. The patient journey starts with the complaints (blue), and can face factors that contribute to a positive patient journey (green) or factors that are perceived as barriers or negative aspects from the patient journey (red). There is also a central factor related to the experience of a multidisciplinary approach for orofacial pain (yellow).

Patients reported that pain and/or trouble in daily functioning usually were the start of the complaints, which caused them to seek help with a healthcare professional. Patients wanted to understand their complaints (what is it, and how can this be resolved?) and receive help to decrease these complaints as quickly as possible.

### 3.4 Primary aim: description of the emotional patient journey: quantitative results

Interestingly, more than half of the patients had a positive experience with the visited healthcare providers, regardless of how many healthcare providers they had visited. However, the majority did not have a satisfactory result throughout their patient journey for the different healthcare providers visited (61.9%–81.4%), or the visited healthcare provider was unable to help the patient (45.0%–57.1%), resulting in visiting another healthcare professional. Another motivation to continue seeking help was that the healthcare provider advised the patient to seek further help (35.0%–57.1%). In some cases, patients continued to seek help from another healthcare provider due to the lack of a personal connection with the healthcare provider (15.0%–57.1%).

### 3.5 Primary aim: description of the emotional patient journey: qualitative results

Even though patients stated they would like to be helped close to home in private clinics instead of specialized clinics, they felt it often was not an option because the expertise was not available. Specialized clinics with a multidisciplinary approach are needed in complex orofacial pain complaints, as there is a team of experts available to help the patient. “*I thought that it was very nice to talk to all professionals during the intake. I had a clear need for help, and the professionals all looked at it from their own expertise, and then they come to you with some advice*.” (Pt04)

### 3.6 Secondary aim: factors contributing to complete patient journey: quantitative results

The number of healthcare providers visited during the patient journey showed an association with readiness to change (*r* = 0.247; *p *= 0.012) and illness perception (*r* = 0.233; *p *= 0.018) in the single regression analyses. A multiple regression analysis was performed, and both readiness to change and illness perception were retained in the model as significant variables (Table 2).

**Table 2.** Single and multiple regression analyses for the association between the primary outcomes “number of healthcare providers” and “patient satisfaction” and different personal factors.

Variable	Single regression analysis		Multiple regression analysis		
	*r*	*p*-value	Standardized beta	*p*-value	Sig. model change
Outcome: Number of healthcare providers					
Locus of Control (Internal)	−0.043	0.666	-	-	-
Locus of Control (External)	0.041	0.684	-	-	-
Locus of Control (Chance)	−0.178	0.073	-	-	-
Illness Perception	0.233	**0.018**	0.200	**0.040**	0.040
Readiness to Change	0.247	**0.012**	0.216	**0.027**	0.012
Catastrophizing	0.022	0.824	-	-	-
Kinesiophobia	0.004	0.968	-	-	-
Outcome: Patient Satisfaction					
Locus of Control (Internal)	−0.212	**0.032**	−0.212	0.032	0.032
Locus of Control (external)	−0.108	0.278	-	-	-
Locus of Control (Chance)	−0.013	0.893	-	-	-
Illness Perception	−0.162	0.103	-	-	-
Readiness to Change	−0.211	**0.033**	-	-	-
Catastrophizing	−0.027	0.787	-	-	-
Kinesiophobia	−0.052	0.603	-	-	-

*r: regression coefficient; sig.: significant. Significant associations are depicted in bold.*

Patient satisfaction of the patient journey was associated with internal locus of control (*r* = −0.212; *p *= 0.032) and readiness to change (*r* = −0.211; *p *= 0.033) in the single regression analysis. The other personal factors (external- and chance-related locus of control, illness perception, catastrophizing and kinesiophobia) did not have an association with patient satisfaction. Using a forward approach, only internal locus of control remained in the model (*r* = −0.212; *p *= 0.032) (Table 2).

### 3.7 Secondary aim: factors contributing to complete patient journey: qualitative results

#### 3.7.1 Factors contributing to a positive patient journey

In some cases, the healthcare provider could not help the patient with their complaints immediately. This wasn’t considered problematic, as long as the healthcare professional was thinking along with the patient and discussed future steps and options for referral. “*I felt like my dentist took me seriously. He did state he could not find any other causes for my problems, and he lengthened my two front teeth to see if it would decrease the pressure. But he understood the powerlessness, and the fact that having so much pain for a long time really has an impact on you*.” (Pt04). These are signs of clear communication and proper soft skills of the healthcare professional, which are seen as helpful for efficient and satisfactory patient journeys. Additionally, these factors contribute to the patients’ understanding of their own complaints, and to a feeling of being taken seriously throughout their journey. Feeling heard was essential to every patient, but very often patients described that specialists did not have enough time and were limited to their own area of expertise, rather than looking at the patient and their problems. When patients do understand their complaints and feel taken seriously, this will eventually lead to more cooperation and success in treatment: “*I think ultimately it is the combination of skills of the healthcare professional [that makes the difference in treatment outcome], but when you have a connection, a certain sensitivity, a click from person to person, you are more receptive to the care being given, and then also to the advice being given*.” (Pt01)

#### 3.7.2 Factors contributing to a negative patient journey

Frequently patients did report that they had to act as their own case manager, where they had to keep asking to be referred when a healthcare professionals could not help them, which was not always easy to do: “*What I find tricky is that as a patient, you are vulnerable in a way as you undergo something you do not quite understand, which is annoying, that hurts and you feel surrendered to someone who knows a lot more about that—not everything—but as a patient you do not always realize that and it is difficult to stay true to yourself and to keep looking for improvement*.” (Pt01). Another challenge was the long wait times to see specialists, especially when they kept being referred from one specialist to another. This was deemed not cost-effective, whereas proper referral and collaboration could reduce overall healthcare costs. Another important financial factor to consider is the insurance coverage, as some patients stated they would not be able to afford the full treatment plan for their orofacial pain if it was not covered by insurance.

What most patients also encountered during their patient journey which made it less efficient, was the perceived lack of knowledge a lot of healthcare professionals had on the complexity of orofacial pain, especially when another disorder makes everything more complex: “*More than that they could not offer. Here it was also very clear that they did not have enough knowledge of my condition. […] Even with someone who is intrinsically motivated they state, ‘I don’t know’*.” (Pt08). This, specifically with the combination of healthcare professionals not listening, resulted in the feeling of helplessness in a lot of patients. “*And then you have to wait it out and see, keep muddling through is what it feels like. That is the annoying thing about it. But yeah, I did that and eventually called for help again, because it was not working*.” (Pt04)

## 4. Discussion

The primary aim of this study was to describe the physical and emotional patient journey of patients with orofacial pain. The secondary aim of this study was to determine which personal factors may have contributed to the experience of patient journey. Through a mixed-methods study design several key outcomes were identified. Even though most of the participants visited three or fewer healthcare providers for their complaints, only 38.2% were satisfied with their patient journey. Factors that contributed negatively to the patient journey experience were healthcare providers not being able to help the patient due to a perceived lack of expertise or knowledge, patients not feeling taken seriously or heard, and long waiting lists.

### 4.1 Triangulation of results: healthcare providers visited

The patient journey of patients with orofacial pain is complex due to different factors. One of the hypotheses was that personal factors would play a role in the journey, for example that highly motivated people would find their way to a specialized center more quickly. One of the personal factors that had an association with the number of healthcare providers visited was indeed readiness to change, which is related to motivation [29]. The other identified personal factor related to the number of healthcare providers visited was illness perception. Patients with a distorted illness perception were more likely to see more healthcare providers, which may be because illness perception may influence treatment outcomes and satisfaction with treatment [37]. When patients are not satisfied, they are more likely to visit another healthcare provider.

The qualitative analysis from the current study showed that patients are often not very satisfied with the professional communication of the visited healthcare providers. Furthermore, even though there was a significant association between the number of healthcare providers visited and patient satisfaction, this association was small. Still, almost half of the patients who had visited relatively few healthcare providers (three or less) were not satisfied with their patient journey. This indicates that other factors are more important for patient satisfaction than the number of healthcare providers visited.

### 4.2 Triangulation of results: patient satisfaction

The quantitative analysis identified an association between internal locus of control and patient satisfaction, as well as an association between readiness to change and patient satisfaction. Furthermore, the qualitative analysis identified external factors such as communication skills from the healthcare professional as very important. Listening to the patients, taking them seriously, and explaining why the healthcare professional can or cannot help them, were all considered just as important as actually getting help. The statements from the quantitative data analysis did show that the reason patients kept seeking help was because healthcare professionals could not help them and advised them to continue seeking help elsewhere.

These results are comparable to other studies [8, 38]. One qualitative study on the care pathways of patients with persistent orofacial pain also showed that these journeys are not always as efficient as patients are looking for help from different healthcare providers to get their pain diagnosed and managed [38]. Patients often have to wait for referrals to specialists or other healthcare providers, and waiting lists are long, which decreases patient journey efficiency and increases frustration [8, 38]. This may explain why even though most of the participants from the current study saw only three healthcare providers, they were still not satisfied with the patient journey.

Furthermore, the qualitative analysis showed that patients often had to ask for referrals themselves and act as their own case manager. Patients stated that they felt general healthcare providers did not have enough knowledge on orofacial pain to properly help or refer them. As orofacial complaints are in the area of the mouth and jaw, half of the patients (49%) visit their general dentist for help at first. Another study shows that when patients ask their dentist for help, but they cannot help them, this can sometimes lead to distrust and disappointment [39]. This feeling of helplessness is a common theme amongst qualitative studies for orofacial pain, including the current study [38, 39, 40]. The current study focused on the patients’ perspective. Previous studies have described the dentists’ perspective and showed that they usually want to help as much as they can, but sometimes lack the knowledge on orofacial pain to properly manage this type of patients [41]. Since these aspects are such an important part to a satisfactory patient journey from a patient’s perspective, future studies should look into the perspective of healthcare providers as well to improve this.

### 4.3 Strengths and limitations

One of the strengths of the current study is the use of the mixed methods design, where over 100 participants filled out a questionnaire on their patient journey and 10 of them were interviewed about their experiences. Even though these 10 participants do not speak for the experience of all 100 participants, we identified some strong themes that were also comparable to themes identified in other studies, indicating that the results are more generalizable for a persistent orofacial pain population [38, 42].

A limitation of the current study is that we only describe the patient journey until patients visit the OPD clinic at ACTA. The OPD clinic at ACTA might not be the last clinic the patients will visit. Some patients were referred to another healthcare provider, so their patient journey continued further. Future studies should also follow the patient journey of those patients, to allow cluster analyses to identify which patients benefit from a specialized clinic such as the OPD clinic, and which patients should be seen by other specialties. Furthermore, only including participants from a single clinic could possibly lead to selection bias, especially since the patients coming to this clinic are often more complex. This should be taken into consideration when interpreting the findings of this study.

Another limitation of this study is that the literacy level of the included participants was not checked, which may have resulted in a bias in the results. Specifically, people with higher levels of literacy tend to be more active in the decision-making process which could also influence the patient journey [43]. Future studies should therefore also take (health) literacy into account when looking at factors influencing patient journeys. Furthermore, the use of questionnaires and surveys regarding experiences in the past could lead to potential recall bias. As most people with orofacial pain start their journey at the dentist or general medical physician, it would be interesting to follow people on their journey from that moment in future research. Lastly, the MPRC questionnaire was translated into Dutch, but not validated in the Dutch language. The English version of the MPRCQ show good validity and responsiveness [29], but as this is not determined for the Dutch version this may influence the interpretation of results.

### 4.4 Clinical implications

Patients in the current study often visited their general dentist as their first healthcare provider for their orofacial complaints but also stated that there was a perceived lack of knowledge on the complexity of orofacial pain and an absence of other healthcare providers who could support the diagnostic and treatment process. Other studies have shown that especially newly graduated dentists do not perceive their knowledge on orofacial pain as adequate [44, 45, 46], which party confirms the perspective of the patients in the current study. It is important to note, however, that the statement on the lack of knowledge was about all healthcare providers visited. The perceived knowledge of other healthcare providers on orofacial pain has not yet been described in the literature. So based on the perspective of the patients of the current study, it is therefore important that the general dentists, as well as other first healthcare providers visited such as the general medical physician or physiotherapist, increase their knowledge on orofacial pain. For general dentists, education regarding orofacial pain should not only be part of the mandatory predoctoral dental curricula as it often is already, but it should be enhanced to the general dentists will experience a higher level of knowledge after graduating. For this, international guidelines to describe the content of such enhanced dental curricula, including some clinical experience with TMD patients as a prerequisite for all dental students before graduation, could be established to ensure higher levels of knowledge on orofacial pain [47].

Furthermore, one of the biggest themes identified that healthcare providers should include in their daily practice, is that the patient wants to be taken seriously. Other studies have also shown that patients with orofacial pain get frustrated when they do not get answers about their complaints, and when healthcare providers do not listen or do not show compassion [40, 42]. Since patients who understand their complaints and who feel that they are taken seriously often establish a stronger therapeutic alliance and show more self-efficacy [48], it is important that healthcare providers take this into consideration when communicating with their patients with orofacial pain.

Lastly, it is important to put the findings of this study in the context of the country and healthcare system in which this study was performed. In the Netherlands, dentists are often the primary point of contact for orofacial pain, whereas in other countries, this may be the maxillofacial surgeon [49]. Hence, the overall results regarding factors that may have influenced the patient journey satisfaction, both from questionnaire data and interviews, should be taken into the clinic and perhaps studied in different countries to confirm the external validity of these results.

## 5. Conclusions

Patients with orofacial pain often directly go to a healthcare provider for help, but experience difficulty finding the right person who can provide them with information, a proper diagnosis, and suitable treatment plan. Many patients are not satisfied with their patient journey, which is not only related to the number of healthcare providers visited, but more so to the emotional experiences during their journey such as being taken seriously and finding someone they feel has enough knowledge to be able to help. Only the personal factors readiness to change, internal-locus of control and chance-related locus of control influence the number of healthcare providers visited or the patient satisfaction, and external factors related to the healthcare providers are important to consider.

## Supplementary Material

Supplementary material associated with this article can be found, in the online version, at https://files.jofph.com/ files/article/1966368637047455744/attachment/ Supplementary%20material.docx.


