# Supplementary material for: Contributing factors for a satisfying orofacial pain patient journey: a mixed-methods study
Source: J Oral Facial Pain Headache. 2025 Sep 12;39(3):84–94. doi: 10.22514/jofph.2025.050 (PMC12520448; doi:10.22514/jofph.2025.050)
Supplement: Supplementary file 1 [file Supplementary-material.docx]

Supplementary material

Supplementary Table 1. Topic Guide (translated from Dutch for publication).

| 1. Opening | □ Explanation of the interview  *Thank you for wanting to partake in this interview. The interview will be recorded, and you can stop any time you want. The interview will be about the patient journey, from the moment you started experiencing complaints in your jaw regio. You have already filled out a questionnaire on this topic, and I would like to dive deeper into some of your answers. Try to answer extensively, and because it is your own experience, there is no right or wrong answer.* |
| --- | --- |
| 2. | ● What was the initial reason to seek help?  ● Who did you first go to for help and why?  ● What were your expectations of this healthcare provider?  ● Did this healthcare provider meet your expectations?  ● What about this healthcare provider contributed to the recovery of your complaints?  ● What about this healthcare provider did not contribute to the recovery of your complaints?  ● Did you seek help after this healthcare provider, and if so, why?  *Questions are repeated for each visited healthcare provider.* |
| 3. | ● When you look back on your patient journey that we just discussed, what did you miss during this process? What advice do you have on how to organize this better?  ● Did you notice anything regarding collaboration between healthcare providers and if so, what did you think? If not, do you think this could have contributed to your recovery?  ● How much did coverage from your health insurance play a role in your decision to go to certain healthcare providers or the ACTA? |
| 4. | Closure  *Is there anything I did not ask, but you would like to discuss? If not, I thank you for your contribution to the study with this interview. I will now stop the recording.* |

ACTA: Academic Centre of Dentistry Amsterdam.

Supplementary Table 2. Overview of healthcare providers visited, the experience and reasons to continue seeking help.

| Number of patients | Number of healthcare providers visited | Top 3 healthcare providers visited | Experience | Reasons to continue seeking help |
| --- | --- | --- | --- | --- |
| 102 | 1 | ● General dentist (49.0%)  ● Orofacial physiotherapist (16.7%)  ● General medical physician/other (10.8%) | ● 57.8% had a positive experience  ● 81.4% did not have a positive result  ● 40.2% were satisfied, 30.4% neutral | ● Healthcare provider could not help me (54.9%)  ● Healthcare provider advised to look for help elsewhere (47.0%)  ● There was no personal connection (32.3%) |
| 58 | 2 | ● Other (31.0%)  ● General dentist (17.2%)  ● Orofacial Pain Dentist (13.8%) | ● 60.3% had a positive experience  ● 69.0% did not have a positive result  ● 55.2% were satisfied, 19.0% neutral | ● Healthcare provider could not help me (53.4%)  ● Healthcare provider advised to look for help elsewhere (48.3%)  ● There was no personal connection (29.3%) |
| 42 | 3 | ● Other (31.0%)  ● Orofacial physiotherapist (23.8%)  ● General dentist (16.7%) | ● 59.5% had a positive experience  ● 61.9% did not have a positive result  ● 47.6% were satisfied, 26.2% neutral | ● Healthcare provider could not help me (52.4%)  ● Healthcare provider advised to look for help elsewhere (42.9%)  ● There was no personal connection (23.8%) |
| 30 | 4 | ● Other (33.3%)  ● Orofacial Pain Dentist (16.7%)  ● Orofacial physiotherapist (13.3%) | ● 73.3% had a positive experience  ● 63.3% did not have a positive result  ● 66.7% were satisfied, 13.3% neutral | ● Healthcare provider could not help me (56.7%)  ● Healthcare provider advised to look for help elsewhere (53.3%)  ● There was no personal connection (16.7%) |
| 20 | 5 | ● Other (40.0%)  ● Orofacial physiotherapist (20.0%)  ● General dentist (15.0%) | ● 75.0% had a positive experience  ● 80.0% did not have a positive result  ● 65.0% were satisfied, 25.0% neutral | ● Healthcare provider could not help me (45.0%)  ● Healthcare provider advised to look for help elsewhere (35.0%)  ● There was no personal connection (15.0%) |
| 15 | 6 | ● Other (40.0%)  ● Orofacial physiotherapist/orofacial pain dentist/general dentist (13.3% each)  ● General medical physician/general physiotherapist/speech therapist (6.7% each) | ● 60.0% had a positive experience  ● 60.0% did not have a positive result  ● 46.7% were satisfied, 46.7% neutral | ● Healthcare provider could not help me (46.7%)  ● Healthcare provider advised to look for help elsewhere (40.0%)  ● There was no personal connection (33.3%) |
| 7 | 7 | ● Orofacial physiotherapist/Neurologist/Other (28.6%)  ● General dentist (14.3%) | ● 57.1% had a positive experience  ● 71.4% did not have a positive result  ● 57.1% were satisfied, 0% neutral | ● Healthcare provider could not help me (57.1%)  ● Healthcare provider advised to look for help elsewhere (57.1%)  ● There was no personal connection (57.1%) |
| 5 | 8 | ● 1 OFP dentist, 1 psychologist, 1 neurologist, 1 general medical physician, 1 other |  |  |
| 2 | 9 | ● 1 speech therapist, 1 general medical physician |  |  |

Each row of this table shows the number of participants (column 1) that visited a certain number of healthcare providers (column 2), which healthcare providers these mostly were (column 3), their experience with these healthcare providers (column 4) and reasons to continue seeking help (column 5).
